# Alpha-ketoglutarate partially alleviates effects of high-fat high-fructose diet in mouse muscle

**DOI:** 10.17179/excli2023-6608

**Published:** 2023-12-05

**Authors:** Myroslava V. Vatashchuk, Maria M. Bayliak, Viktoriia V. Hurza, Oleh I. Demianchuk, Dmytro V. Gospodaryov, Volodymyr I. Lushchak

**Affiliations:** 1 Department of Biochemistry and Biotechnology, Vasyl Stefanyk Precarpathian National University, 57 Shevchenko Str., Ivano-Frankivsk, 76018, Ukraine; 2 Research and Development University, 13a Shota Rustaveli Str., Ivano-Frankivsk, 76018, Ukraine

**Keywords:** high-fat high-fructose diet, mouse, energy metabolism, glycolysis, lipotoxicity

## Abstract

Consumption of high-calorie diets leads to excessive accumulation of storage lipids in adipose tissue. Metabolic changes occur not only in adipose tissue but in other tissues, too, such as liver, heart, muscle, and brain. This study aimed to explore the effects of high-fat high-fructose diet (HFFD) alone and in the combination with alpha-ketoglutarate (AKG), a well-known cellular metabolite, on energy metabolism in the skeletal muscle of C57BL/6J mice. Five-month-old male mice were divided into four groups - the control one fed a standard diet (10 % kcal fat), HFFD group fed a high-fat high-fructose diet (45 % kcal fat, 15 % kcal fructose), AKG group fed a standard diet with 1 % sodium AKG in drinking water, and HFFD + AKG group fed HFFD and water with 1 % sodium AKG. The dietary regimens lasted 8 weeks. Mice fed HFFD had higher levels of storage triacylglycerides, lower levels of glycogen, and total water-soluble protein, and higher activities of key glycolytic enzymes, namely hexokinase, phosphofructokinase, and pyruvate kinase, as compared with the control group. The results suggest that muscles of HFFD mice may suffer from lipotoxicity. In HFFD + AKG mice, levels of the metabolites and activities of glycolytic enzymes did not differ from the respective values in the control group, except for the activity of pyruvate kinase, which was significantly lower in HFFD + AKG group compared with the control. Thus, metabolic changes in mouse skeletal muscles, caused by HFFD, were alleviated by AKG, indicating a protective role of AKG regarding lipotoxicity.

## Abbreviations

ADP/ATP adenosine diphosphate/triphosphate

EDTA ethylenediaminetetracetic acid

FK fructokinase

HFD high fat diet

HFFD high-fat high-fructose diet

HK hexokinase

NAD(P)^+^/

NAD(P)H nicotinamide adenine dinucleotide (phosphate) reduced/oxidized

PFK phosphofructokinase

PK pyruvate kinase

PMSF phenylmethylsulfonyl fluoride

TAG triacylglycerides

## Introduction

The obesity and related metabolic complications have become a widespread problem in Western society and developing countries. The rising incidence of obesity can be largely attributed to the increased availability and consumption of high-calorie foods and insufficient physical activity (Sishi et al., 2011[45]; Vatashchuk et al., 2022[52]). Excessive intake of calories, that exceeds energy expenditures, triggers unfavorable changes in the body's metabolism. In particular, the synthesis and accumulation of storage lipids is intensified in adipose tissue that leads to body mass gain. Moreover, fat accumulation is accompanied by development of oxidative stress and inflammation in adipose and other tissues (Lechuga-Sancho et al., 2018[31]). Metabolic changes occur not only in adipose tissue, but in other tissues, too, such as liver, heart, muscle, and brain (Bhatt et al., 2006[8]; Lepczynski et al., 2021[33]; Bayliak et al., 2022[7]). In addition, in obese animals and humans, fat is accumulated not only in adipose tissue but also other organs (*e.g*., liver, muscle) (van Herpen and Schrauwen-Hinderling, 2008[50]; Denies et al., 2014[16]).

Skeletal muscles make up the bulk of body mass in humans and other mammals and perform various physiological functions, that require high energy expenditures (Abrigo et al., 2016[1]). Several studies have shown that high-calorie diets rich either in fats or in both fats and carbohydrates, can cause muscle loss, atrophy, and dysfunction of skeletal muscles in model animals (Bhatt et al., 2006[8]; Sishi et al., 2011[45]; Spooner et al., 2021[47]). In particular, high-fat diet (HFD) and high-fat high-sucrose diet were found to induce myodegeneration (Sishi et al., 2011[45]; Abrigo et al., 2016[1]; Rasool et al., 2018[40]). Accordingly, muscle loss can be manifested not only in physical dysfunction but also in metabolic disorders. Skeletal muscle mass is maintained by adjusting the synthesis and breakdown of muscle protein (Andreou and Tavernarakis, 2010[2]). It has been shown that a long-term high-fat diet causes atrophy of various leg muscles in mice, changing the distribution between slow oxidative and fast glycolytic muscle fibers in favor of the latter (Denies et al., 2014[16]; Abrigo et al., 2016[1]). In addition, mice on HFD tend to accumulate more fat in muscles, that can disrupt normal muscle function and impair energy metabolism (Goto-Inoue et al., 2013[21]; Martinez-Huenchullan et al., 2018[35]).

In our previous study, we found that a diet high in both fat and fructose (high-fat high-fructose diet, HFFD) activated fructolysis and glycolysis, led to accumulation of glycogen, and induced inflammation and oxidative stress in the liver of mice (Bayliak et al., 2022[7]). Our study also showed that the metabolic effects of HFFD in the liver were attenuated when HFFD was supplemented with α-ketoglutarate (AKG) (Bayliak et al., 2022[4]), which is an important intermediate metabolite in the Krebs cycle and plays a crucial role in many metabolic processes in animals and humans (Bayliak and Lushchak, 2021[5]). 

A specific feature of the liver is that it can metabolize fructose due to the presence of the enzyme fructokinase. This enzyme is absent in other organs like muscle and adipose tissue (Ishimoto et al., 2012[25]). In muscles and adipose tissue, fructose can be utilized by hexokinase. At the same time, glucose is a more favorable substrate for hexokinase; therefore, it competitively inhibits fructose phosphorylation by hexokinase (Varma et al., 2015[51]). Hence, HFFD may cause different effects on the metabolism of different organs. In skeletal muscles, the main energy substrates are muscle glycogen, blood glucose, and fatty acids derived from both intramuscular triacylglycerides and adipose tissue triacylglyceride stores (Hargreaves and Spriet, 2020[23]). We supposed that metabolic response of mouse ske-letal muscles to HFFD would differ from that of mouse liver. Hence, the current work is a continuation of the previous study on mouse liver (Bayliak et al., 2022[7]) and aims to investigate the effects of HFFD and AKG separately and in combination on some parameters of energy metabolism such as accumulation of storage lipids and glycogen, and activity of key glycolytic enzymes, namely hexokinase (HK), phosphofructokinase (PFK), and pyruvate kinase (PK), in the mouse skeletal muscles. Given that muscles have lower abi-lity to metabolize fructose (a nutritional component of HFFD), we expected that muscles of HFFD-mice would rely mostly on triacylglycerides as an energy source. In addition, we assumed that HFFD would lead to fat accumulation and protein loss, and weakness of skeletal muscles as it was observed in previous similar studies (Sishi et al., 2011[45]; Denies et al., 2014[16]; Abrido et al., 2016[1]; Rasool et al., 2018[40]). In view of the involvement of AKG in the biosynthesis of amino acids and improvement of muscle recovery during injuries (Harrison and Pierzynowski, 2008[24]; Dobrowolski et al., 2013[17]; Wang et al., 2016[53]; Bayliak and Lushchak, 2021[5]) we supposed that AKG supplementation may prevent metabolic changes such as protein loss in HFFD-fed mice.

## Materials and Methods

### Reagents

ADP, aldolase, amyloglucosidase, ATP, bovine serum albumin, di-sodium hydrogen phosphate (Na_2_HPO_4_), fructose-6-phosphate, glycerol-3-phosphate dehydrogenase, glucose, glucose-6-phosphate, glucose-6-phosphate dehydrogenase, imidazole, magnesium chloride hexahydrate (MgCl_2_×6H_2_O), phosphoenolpyruvate, potassium chloride (КСl), potassium dihydrogen phosphate (KH_2_PO_4_), sodium azide (NaN_3_), sodium chloride (NaCl), sodium pyruvate, triose-phosphate isomerase and were purchased from Sigma-Aldrich (USA). NAD^+^, NADH, NADP^+^, NADPH, EDTA, phenylmethylsulfonyl fluoride (PMSF), dithiothreitol, sodium fluoride (NaF), lactate dehydrogenase, Coomassie Brilliant blue G-250 and Triton X-100 were purchased from Carl Roth (Karlsruhe, Germany). Diagnostic kits for determination of glucose and triacylglycerides were from Private Joint-Stock Company «Reagent» (Dnipro, Ukraine). Alpha-ketoglutaric acid was from Protista AB (Sweden). 

### Animals and feeding regimes

In this study, we used C57BL/6 mice as a well-known model of diet-induced obesity (Collins et al., 2004[14]; Chu et al., 2017[13]; Siersbæk et al., 2020[44]; Casimiro et al., 2021[11]). Previous studies showed that male mice are more susceptible to high-calorie diet, when fed from 4-8 weeks of age, which corresponds to 14-18 years of age in humans (Chu et al., 2017[13]; Casimiro et al., 2021[11]). We were interested in whether sexually mature young male mice (about 5-6 months), equivalent to humans aged 25 to 30 years (Flurkey et al., 2007[18]), are as susceptible to dietary interventions as adolescent mice. For the experiment, five-month-old male mice were randomly divided into four groups. Each group consisted of 12-14 mice (2-3 mice per cage, 4-5 cages per group). The control group was fed a standard rodent diet (10 kcal % fat) containing 21.8 % protein, 4.8 % fat, 69.1 % carbohydrates, and 3.9 % fiber (“Rezon-1”, Kyiv, Ukraine). The experimental groups of mice had different feeding regimens. The AKG group received the standard diet and 1 % AKG in drinking water. First, a stock 10 % solution of the sodium salt of α-ketoglutaric acid (Protista AB, Sweden) was prepared, which was then 10-fold diluted in drinking water to obtain a concentration of AKG of 1 %. Тhe HFFD group was fed a high-fat high-fructose diet (45 kcal % fat, 15 kcal % fructose, and 10 % protein). The HFFD was prepared manually by mixing all components (per 1 kg: 250 g lard, 550 g standard chow, 200 g fructose and 10 ml bile as an emulsifier) and then divided into small pellets. The high-calorie diet containing 45 % calories from fat and supplemented with fructose is one the diets commonly used to study metabolic disorders and obesity in mice (Siersbæk et al., 2020[44]; Casimiro et al., 2021[11]). The HFFD + AKG group received the high-fat high-fructose diet and drinking water with 1 % sodium AKG solution. The food was changed every day for HFFD group and HFFD+AKG group. In the control group and AKG group food was changed weekly. All groups of mice had unlimited access to food and water. The mice were kept on the corresponding diets for the next eight weeks under a 12-h light/dark cycle (6 a.m./6 p.m.) at 22 ± 2°C temperature, and 50-60 % humidity (Bayliak et al., 2022[7]). All experimental protocols were approved by the Animal Experimental Committee of Vasyl Stefanyk Precarpathian National University and were conducted in accordance with the Directive 2010/63/EU of the European Parliament and of the Council of 22 September 2010 on the protection of animals used for scientific purposes.

### Tissue collection

Before sampling, mice were fasted from 5:00 pm to 9:00 am. Mice from each experimental group were divided randomly into two subgroups. One subgroup was euthanized using light carbon dioxide anesthesia and used for collecting skeletal muscles. Another group was euthanized by cervical dislocation on ice and was used in other experiments (Bayliak et al., 2022[7]). After sampling, skeletal muscles were frozen quickly in liquid nitrogen and then stored at -80 °C. Dissection of muscles was made in such a way that the sample contained mainly the *tibialis anterior* and *extensor digitorum longus* with minor amount of *muscle soleus*. After freezing, the samples were used to determine various biochemical parameters. To determine a specific group of parameters, a portion of the frozen tissue was taken and further homogenized according to the appropriate protocols as described below.

### Tissue homogenization and assays of enzyme activities

To determine the activities of glycolytic enzymes, frozen tissue samples were homo-genized in 50 mM imidazole buffer (pH 7.5) containing 0.5 mM EDTA, 1 mM PMSF, 1 mM dithiothreitol, 20 mM NaF, and 150 mM KCl. Homogenates were then centrifuged (16 100 × g, 15 min, 4 °C) and supernatants were collected for biochemical measurements.

Activities of glycolytic enzymes, hexokinase (HK), phosphofructokinase (PFK), and pyruvate kinase (PK) were measured spectrophotometrically in coupled reactions as described earlier (Lushchak et al., 1998[34]; Sorochynska et al., 2019[46]). The HK activity was measured by monitoring NADP^+^ reduction at 340 nm in the reaction mixture consisting of 50 mM imidazole buffer (pH 7.5), 10 mM glucose, 5 mM MgCl_2_, 2 mМ ATP, 0.2 mМ NADP^+^, 0.5 U glucose-6-phosphate dehydrogenase (#G7877, Sigma-Aldrich), and 30 µl of supernatant in the volume of 1 ml. The PFK and PK activities were measured by monitoring NADH oxidation at 340 nm. Reaction mixture for PFK activity measurement consisted of 50 mM imidazole buffer (pH 7.5), 5 mM fructose 6-phosphate, 5 mM MgCl_2_, 5 mМ ATP, 0.16 mМ NADH, 50 mM КСl, 0.5 U aldolase (#А1893, Sigma-Aldrich), 0.5 U triose-phosphate isomerase (#Т2391, Sigma-Aldrich), 2 U glycerol-3-phosphate dehydrogenase (#10127752001, Sigma-Aldrich) and 5 µl of supernatant in the final volume of 1 ml. Reaction mixture for PK activity measurement contained 50 mM imidazole buffer (pH 7.5), 1 mM phospho-enolpyruvate, 5 mM MgCl_2_, 50 mM КСl, 2.5 mM ADP, 0.16 mM NADH, 2.5 U lactate dehydrogenase (#6060.1, Carl Roth), and 2 µl of supernatant in the final volume of 1 ml. One unit of enzyme activity is defined as the amount of the enzyme consuming 1 μmol of substrate or generating 1 μmol of product per minute; activities are expressed as international units per milligram of soluble protein (U/mg protein).

Soluble protein content was determined with the Coomassie Brilliant blue G-250 with bovine serum albumin as a standard (Bradford, 1976[9]).

### Tissue homogenization and assays of metabolite levels

For measurement of glucose and glycogen levels, frozen muscle tissues were homogenized in 50 mM potassium phosphate buffer in a 1:10 w:v ratio. To determine triacylglyceride (TAG) levels, samples were homogenized in phosphate buffered saline (10 mM Na_2_HPO_4_, 2 mM КН_2_РО_4_, 137 mМ NaCl, 2.7 mM KCl) with 1 % Triton X-100 in a 1:10 w:v ratio. All homogenates were then heated at 70 °C to inactivate cellular lipases and glycosidases and, after cooling, centrifuged (12 000 × g, 10 min, 21 °C). Free glucose and TAG levels were measured using appropriate diagnostic kits (Private Joint-Stock Company «Reagent», Dnipro) according to the manufacturer's recommendations. To determine glycogen, supernatants were incubated with amyloglucosidase (#10115, Sigma-Aldrich; 0.56 U/μL) for 4 h at 37 °C. Glycogen level was calculated as the difference between glucose levels before (free glucose) and after incubation with amyloglucosidase (free glucose plus glucose produced by the breakdown of glycogen). Results are expressed as micrograms glucose or TAG per gram of wet muscle mass.

### Statistical analysis

Data visualization was performed using GraphPad Prism (version 8.01). Statistical analysis of the results was performed using R software (version 4.2.2). Data were subjected to one-way analysis of variance followed by Duncan's new multiple range test implemented in R (package DescTools). The data are presented as mean ± standard error of the mean (SEM). The difference between groups, for which *P*-value calculated by Duncan's test was less than 0.05, was considered to be statistically significant.

## Results

### Levels of metabolites

**Figure 1:**
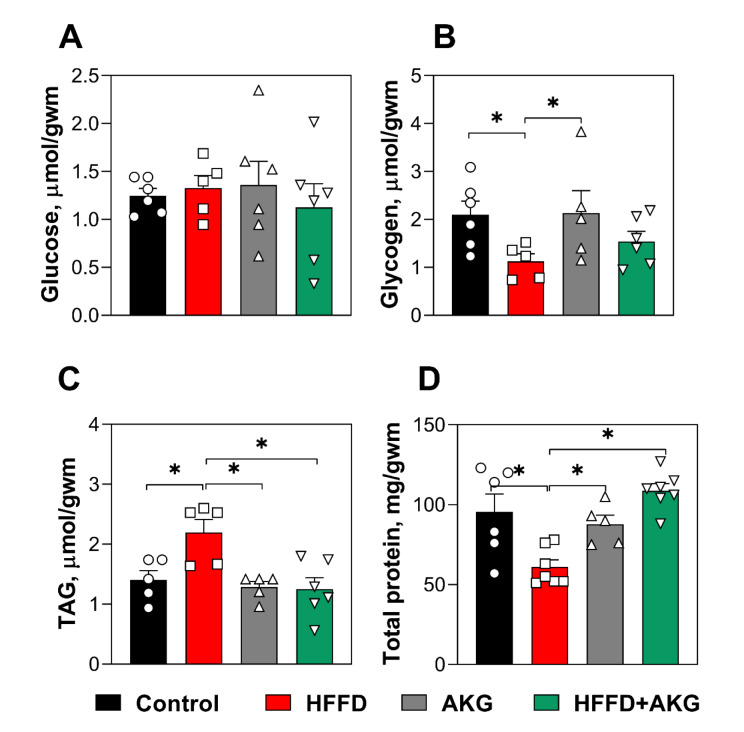
Metabolite levels in the skeletal muscles of C57BL/6J mice fed standard food (control group), high-fat high-fructose diet (HFFD group), standard food with 1 % AKG in the drinking water (AKG group) or high-fat high-fructose diet plus 1 % AKG in the drinking water (HFFD + AKG group) over eight weeks. Levels of glucose (A), glycogen (B), triacylglycerides (TAG, C), and water-soluble protein (D). Data are presented as mean ± standard error of the mean (SEM) from 4-7 mice in each group. *Significant difference (whether *P* < 0.05) between groups was evaluated by Duncan's new multiple range test: (B) control vs. HFFD, p=0.03987; AKG vs HFFD, p=0.0472; (C) control vs. HFFD, p= 0.0063, AKG vs HFFD, p= 0.0031; HFFD vs. HFFD+AKG, p=0.0020

None of the dietary regimens affected free glucose levels in the muscles of the mice (Fi-gure 1A(Fig. 1)). Glycogen content in the muscles of mice fed HFFD was 52 % lower than in the control and AKG groups (Figure 1B(Fig. 1)). Only in the muscles of mice fed HFFD, TAG level was about 36 % higher, than in other groups, whereas TAG levels in other experimental groups virtually corresponded ones in the muscles of the control group (Figure 1C(Fig. 1)). Total levels of water-soluble protein were also affected only by HFFD. In particular, levels of water-soluble protein were 38 % lower in muscles of HFFD-fed mice then in the control group (Figure 1D(Fig. 1)).

### Activities of key glycolytic enzymes

**Figure 2:**
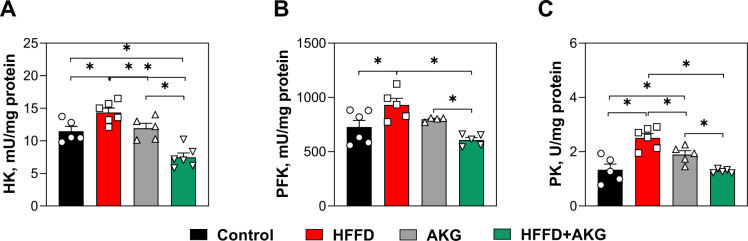
The activity of key glycolytic enzymes in the skeletal muscles of C57BL/6J mice fed standard food (control group), high-fat high-fructose diet (HFFD group), standard food with 1 % AKG in the drinking water (AKG group) or high-fat high-fructose diet plus 1 % AKG in the drinking water (HFFD + AKG group) over eight weeks. Activities of hexokinase (HK, A), phosphofructokinase (PFK, B), and pyruvate kinase (PK, C). Data are presented as mean ± SEM from 4-7 mice in each group. *Significant difference (*P* < 0.05) between groups was evaluated by Duncan's new multiple range test: (A) control vs. HFFD, p=0.01351; control vs. HFFD+AKG, p=0.0009; AKG vs. HFFD, p=0.0279; AKG vs. HFFD+AKG, p=0.00046; HFFD vs. HFFD+AKG, p<0.0001; (B) control vs. HFFD, p=0.01104; AKG vs HFFD+AKG, p=0.02729; HFFD vs. HFFD+AKG, p=0.0006; (C) control vs. HFFD, p= 0.00005; control vs. AKG, p= 0.0217; AKG vs HFFD; p= 0.00994; AKG vs. HFFD+AKG; p= 0.01712; HFFD vs. HFFD+AKG, p= 0.00004.

The activities of three key glycolytic enzymes, namely HK, PFK and PK, showed similar trends in mouse muscles under differ-rent dietary regimens (Figure 2(Fig. 2)). In HFFD-fed mice, the activity of HK, PFK, and PK was 20 %, 22 %, and 47 %, higher, respectively, compared to the control group (Figure 2A-C(Fig. 2)). In the AKG group, the activities of HK and PFK did not differ from the control values (Figure 2A-B(Fig. 2)), whereas PK activity was 30 % higher than that in the control group (Figure 2C(Fig. 2)). HK activity was lower by 35 % and 48 % in the muscles of mice fed HFFD + AKG than in the control and HFFD-fed ones, respectively (Figure 2A(Fig. 2)). The activities of PFK and PK showed similar values in the control group and HFFD + AKG group. However, PFK and PK activities were lower in mice fed HFFD + AKG when comparing with the AKG group and HFFD group. In particular, the activities of HK, PFK, and PK were 47 %, 38 %, and 47 % lower, respectively, in the HFFD + AKG group than in mice fed HFFD only.

## Discussion

### Feeding with HFFD stimulates glycolysis and accumulation of triacylglycerides in mouse skeletal muscles

Skeletal muscle is the tissue that uses most of the organism's energy during exercise. The energy required for muscle contraction, protein synthesis, and other cellular processes is provided by different energy sources. The choice of energy source depends on the muscle type, intensity and duration of the physical activity. Short bursts of intense activity rely on ATP and creatine phosphate, while longer, less intense activities utilize glycogen and fatty acids, derived from both intramuscular triacylglycerides and adipose tissue triacylglyceride stores (Barclay, 2017[3]; Hargreaves and Spriet, 2020[23]). Glycogen, which is a form of glucose storage in mammals, can support both anaerobic and aerobic activities, whereas fats can only be metabolized aerobically (Jensen et al., 2011[26]; Urschel and McKenzie, 2021[49]). Moreover, skeletal muscle is one of the tissues where insulin stimulates glucose uptake to remove glucose from the blood, and the absorbed glucose is incorporated into glycogen (Jensen et al., 2011[26]).

This study showed that HFFD had no effect on free glucose levels but decreased glycogen levels in the mouse skeletal muscles. In addition, higher activities of glycolytic enzymes, higher TAG levels and lower total protein were detected in the muscles from mice fed HFFD.

**Figure 3:**
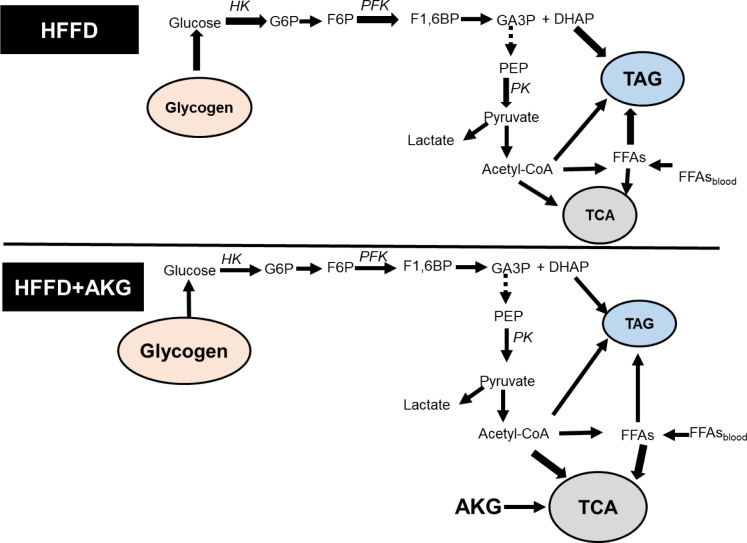
Effects of HFFD and AKG on energy metabolism in mouse skeletal muscle. HFFD leads to glycogen depletion, activation of glycolysis and accumulation of triacylglycerides in the muscle. Supplementation with AKG prevents metabolic alterations caused by HFFD. Bold lines denote the activation of the process. *Abbreviations*: HK - hexokinase, G6P - glucose-6-phosphate, F6P - fructose-6-phosphate, F1,6BP - fructose-1,6-biphosphate, PFK - phosphofructokinase, DHAP - dihydroxyacetone phosphate, GA3P - glyceraldehyde-3-phosphate, PEP - phosphoenolpyruvate, PK - pyruvate kinase; FFAs - free fatty acids, TCA - tricarboxylic acid cycle, TAG - triacylglycerides.

Previously, higher muscle TAG content was observed in mice fed a high-fat diet (HFD) (Goto-Inoue et al., 2013[21]; Martinez-Huenchullan et al., 2018[35]). Muscle lipid accumulation and/or changes in lipid metabolism are supposed to be involved in the development of glucose intolerance in animals fed HFD (van Herpen and Schrauwen-Hinderling, 2008[50]). It has been shown that the development of insulin resistance in muscles is accompanied by glycogen depletion, lipid accumulation, and impaired normal tissue function (Goto-Inoue et al., 2013[21]; Rachek, 2014[38]). Our results indicate that the diet rich in both fats and fructose leads to similar results to those observed in HFD mice, namely it decreased glycogen levels and increased TAG accumulation. HFFD might stimulate lipid flow to skeletal muscle, leading to reduced oxidation of fatty acids and enhanced lipid accumulation in skeletal muscle that could contribute to insulin resistance. In support of this, the study on rats found that feeding with HFFD resulted in higher levels of TAG and ceramide (Crescenzo et al., 2015[15]). The study of Crescenzo et al. (2015[15]) also found that HFFD led to a lower mitochondrial energetic efficiency in skeletal rat muscles. In our experiment, HFFD could also worsen operation of mitochondria. Lowered mitochondrial efficiency can explain higher activity of glycolytic enzymes in the skeletal muscles of HFFD-fed mice. Namely, to compensate for mitochondrial dysfunction, namely inability to effectively produce energy in the form of ATP aerobically, muscle cells activate glycolytic anaerobic pathway to obtain energy. Since fatty acids from triacylglycerides can only be utilized aerobically, the use of lipids as an energy source may decrease, and muscle cells actively breakdown glycogen to provide glucose as energy substrate (Figure 3(Fig. 3)). As a result, it can lead to glycogen depletion in the muscles of HFFD-fed mice (Figure 1B(Fig. 1)). Lower glycogen levels in the muscles of HFFD-fed mice also suggest that the inability to effectively metabolize fructose due to the lack of fructokinase, which is mainly expressed in the liver (Ishimoto et al., 2012[25]). In muscles, fructose can be metabolized by hexokinase but glucose may hinder phosphorylation of fructose by hexokinase (Varma et al., 2015[51]). 

Pyruvate, which is produced in glycolysis, can further undergo oxidative decarboxylation to acetyl-CoA by the pyruvate dehydrogenase complex or be converted to lactate by lactate dehydrogenase. Pyruvate dehydrogenase, the main enzyme of pyruvate dehydrogenase complex, can be inhibited by PDH kinase (PDK) isoforms, thereby decreasing acetyl-CoA supply into the TCA cycle or to the fatty acid biosynthesis (Leblanc et al., 2008[30]; Rinnankoski-Tuikka et al., 2012[41]; Zhang et al., 2014[58]; Bayliak et al., 2022[7]). Moreover, PDK4 isoform, that is highly expressed in skeletal muscle, is up-regulated on HFD and shows frequently elevated levels in diabetic individuals (Leblanc et al., 2008[30]; Rinnankoski-Tuikka et al., 2012[41]). Given that, we can suppose that activation of glycolytic enzymes in muscles of HFFD-fed mice can be also connected with PDK-mediated inhibition of oxidative metabolism of glucose. The higher TAG levels in muscles of HFFD-fed mice can be connected with elevated TAG levels in the blood of these mice (Bayliak et al., 2022[7]). To compensate for glucose deficiency, muscle cells actively absorb lipids from the blood, but due to impaired mitochondrial respiration, it seems that TAGs are not actively used as a source of energy, but rather accumulate as fat depots. 

Insulin resistance is mostly accompanied by hyperglycemia (Lee et al., 2017[32]), but in the previous study, we did not detect any differences in fasting blood glucose levels between mice fed standard diet and HFFD (Bayliak et al., 2022[7]). It was shown, that in some cases, fasting glucose levels may not be elevated in HFD-fed rodents (Siersbæk et al., 2020[44]), when certain compensatory mechanisms can be induced. In particular, pancreas produces more insulin to compensate insensitivity to it (Shinozaki et al., 1996[43]).

Mice fed HFD also developed typical signs of muscle wasting, such as weakness, loss of muscle mass, and decreased fiber diameter (Abrigo et al., 2016[1]). Here, we observed that HFFD led to a lower total protein level in the mouse skeletal muscles, that can reflect a loss of muscle mass. It was suggested that a loss of muscle mass may be related to the development of insulin resistance (Wang et al., 2006[54]). In turn, insulin resistance may lead to muscle wasting via attenuation of PI3K/Akt signaling, leading to activation of caspase-3 and the ubiquitin-proteasome proteolytic pathway, and triggering muscle protein degradation (Wang et al., 2006[54]). An increase in proportion of skeletal muscle fibers with glycolytic phenotype (Denies et al., 2014[16]; Abrigo et al., 2016[1]) can also contribute to atrophy of various leg muscles in mice fed a high-fat diet during a long period.

Generally, decrease in protein levels, accumulation of TAGs, depletion of glycogen, and activation of glycolysis in muscles of HFFD-fed mice can be considered as signs of insulin resistance development.

### Alpha-ketoglutarate prevents HFFD-induced metabolic changes in mouse muscle

In this study, we have established causal relationship between dietary regimen and studied metabolic parameters. We found that feeding with HFFD with AKG abrogated the increase in the activities of glycolytic enzymes, TAG accumulation, glycogen depletion and the decrease in protein levels in the mouse muscles. Such effects of AKG can be explained by its direct involvement in ATP production and its signaling or regulatory function in different processes. As a cellular intermediate, AKG is a substrate of glutamate dehydrogenase and aminotransferases and can be directly involved in amino acid synthesis (Harrison and Pierzynowski, 2008[24]; Bayliak et al., 2016[6]; Chen et al., 2018[12]). In addition, dietary AKG was shown to stimulate amino acid uptake in model animals (Lambert et al., 2006[29]). Via increasing amino acid levels, AKG may activate the mechanistic target-of-rapamycin (mTOR) kinase, which is one of the major regulators of anabolic processes, in particular protein biosynthesis (Wang et al., 2016[53]; Bayliak and Lushchak, 2021[5]). Some studies have reported a potential role of AKG in protecting against muscle atrophy during exercise, showing that AKG could prevent protein degradation via different signaling pathways, in particular, via the inhibition of expression of enzymes related to proteolysis (Cai et al., 2018[10]) and stimulation of adrenaline secretion by 2‐oxoglutarate receptor 1 (OXGR1) expressed in adrenal glands (Yuan et al., 2020[57]). These mechanisms seem to contribute to the effects of AKG on the maintenance of total protein levels in the muscles of the HFFD + AKG group. It cannot be excluded that the prevention of intramuscular lipid accumulation and glycogen depletion in HFFD + AKG group compared with HFFD group can also be connected with AKG-mediated production of adrenaline. Adrenaline is known to bind to both the lipolytic β-adrenergic receptors and the antilipolytic α2-adrenergic receptors. The balance between activation of the α2- and β-receptors determines the overall level of lipolysis within a tissue (Schmidt et al., 2014[42]). In skeletal muscle cells, adrenergic stimulation through β2-adrenoreceptors can increase glycogen synthesis (Yamamoto et al., 2007[56]). Thus, lipid accumulation and glycogen depletion, observed in HFFD + AKG mice, can likely be connected with the activation of β-adrenoreceptor-related signaling pathways. In addition, it was demonstrated earlier that AKG induced beige adipogenesis in HFD mice, thus preventing the obesity development (Tian et al., 2020)[48]. This mechanism can also contribute to the protective effects of AKG in HFFD-fed mice in our experiments; in particular, AKG might prevent intramuscular lipid accumulation via stimulation of proliferation of brown adipose tissue.

Metabolic changes in the muscles caused by HFFD are themselves indicative of the development of insulin tolerance. In turn, muscles of HFFD + AKG mice do not demonstrate similar signs of insulin insensitivity. It was also shown earlier that AKG or its analogues may stimulate insulin secretion (Rabaglia et al., 2005[37]; Willenborg et al., 2009[55]). Insulin was shown to suppress expression of transcription factor Nrf2 (nuclear factor erythroid 2-related factor 2) via mTOR signaling pathway (Ghosh et al., 2017[20]). In turn, insulin was shown to induce mTOR signaling pathway (Rapley et al., 2011[39]; Muta et al., 2015[36]). Nrf2 protein is one of the regulators of expression of glycolytic enzymes, and the inhibition of Nrf2 was found to decrease activities of glycolytic enzymes in model animals (Fu et al., 2019[19]; Bayliak et al., 2020[4]). We observed that feeding HFFD with AKG abrogated the increase in the activities of glycolytic enzymes observed in HFFD mice. Even more, activity of HK, an enzyme that phosphorylates glucose, was lower in HFFD + AKG group than in the control mice. In addition, PDK4 is one of the Nrf2 targets. It was found earlier, that the PDK4 inhibition may alleviate some symptoms of type 2 diabetes in mice; in particular, PDK4 deficiency was found to decrease blood glucose and improved glucose tolerance and insulin sensiti-vity in mice with diet-induced obesity (Jeoung and Harris, 2008[27]). Inhibition of Nrf2 via insulin signaling might promoted lower PDK4 levels that may result in the activation of pyruvate dehydrogenase complex and improving oxidative metabolism in HFFD+AKG-fed mice. Together, our results support AKG-mediated improvement of insulin signaling followed by Nrf2 inhibition in the muscle of HFFD + AKG mice. 

Insulin resistance in muscles can significantly affect other organs and overall health status of the organism. In particular, insulin resistance can also promote the release of free fatty acids from adipose tissue into the bloodstream, contributing to high triglyceride le-vels and an increased risk of cardiovascular diseases such as heart disease, hypertension, and atherosclerosis (Shinozaki et al., 1996[43]; Khafagy and Dash, 2021[28]). Increased TAG levels found in HFFD-fed muscles can confirm lipotoxicity associated with insulin resistance. At the same, revealed ameliorating effects of AKG on signs of insulin resistance in mouse muscles make this compound a perspective natural candidate for management of insulin resistance. Our results are consistent with recent studies which demonstrated the use of AKG in the treatment of certain diseases associated with metabolic disorders in model organisms (Dobrowolski et al., 2013[17]; Wang et al., 2016[53]; Tian et al., 2020[48]; Bayliak et al., 2022[7]). There are few studies examining the potential beneficial effects of AKG supplements on the humans. However, the human studies confirm the effectiveness of AKG in muscle growth, wound healing, and faster recovery after surgery (Harrison and Pierzynowski, 2008[24]; Gyanwali et al., 2022[22]). Therefore, we can surmise that AKG could potentially have therapeutic effects in humans with obesity and insulin resistance; however, the role of AKG in cellular metabolism in regulation of insulin-dependent metabolic responses needs to be studied in more detail.

It should be noted that the standard food supplemented with AKG (AKG group) did not significantly affect the studied biochemical parameters in the muscles, except increasing of PK activity. In our previous study, we also did not observe a significant influence of AKG (added to the standard food) on liver energy metabolism. However, livers of the AKG-fed mice had higher activity of the antioxidant defense enzymes as compared to the control mice (Bayliak et al., 2022[7]). It seems that HFFD modulates the effects of AKG, and *vice versa*, and in healthy individuals, AKG acts mostly as a hormetin, improving operation of antioxidant defense systems. 

## Conclusions

This study demonstrates that an eight-week feeding with a high-fat high-fructose diet induces conspicuous metabolic alterations in the skeletal muscles of male mice. The changes include accumulation of triacylglycerides, decrease in the levels of glycogen and total protein, and increase in the activities of key glycolytic enzymes. Together, these results suggest that the muscles of HFFD mice show signs of insulin insensitivity probably resulting from lipotoxicity. Supplementation of HFFD with AKG alleviated all metabolic changes observed in the muscle of HFFD mice (Figure 3(Fig. 3)). This indicates an ameliorative role of AKG regarding lipotoxicity in murine skeletal muscles. Our results suggest that AKG is a perspective natural candidate for management of insulin resistance and related metabolic complications. Previous studies on humans were focused mainly on such effects of AKG as reduction of muscle protein degradation and bone mineral loss and restoration against surgeries. However, it seems that therapeutic effects of AKG can be broader. Taking into account, that no major adverse effects of AKG supplementation have been reported (Gyanwali et al., 2022[22]), further preclinical and clinical studies can help to better understand the role of AKG in the prevention and treatment of human metabolic diseases. 

## Notes

Maria M. Bayliak and Volodymyr I. Lushchak (Department of Biochemistry and Biotechnology, Vasyl Stefanyk Precarpathian National University, 57 Shevchenko Str., Ivano-Frankivsk, 76018, Ukraine; E-mail: volodymyr.lushchak@pnu.edu.ua) contributed equally as corresponding author.

## Declaration

### Acknowledgments

We thank our student Kateryna Starchevska for technical assistance with biochemical measurements. This research was supported by a grant from National Research Foundation of Ukraine (#2020.02/0118) to MMB.

### Conflict of interest

The authors declare that they have no conflict of interest.

### Ethical statements

All mouse protocols were approved by the Animal Experimental Committee of Vasyl Stefanyk Precarpathian National University (Ukraine) and were conducted in accordance with the European Union for the protection of animals used for scientific purposes of 22 September 2010 (2010/63/EU)*. *The current study complies with the ARRIVE Guidelines for reporting *in vivo* experiments (https://arriveguidelines.org/arrive-guidelines).

### Authorship contribution statement

Myroslava Vatashchuk: Investigation, data curation, validation and visualization, writing of original draft; Maria Bayliak: Conceptualization, supervision, writing of original draft, review & editing, funding acquisition; Viktoriia Hurza and Oleh Demianchuk: Investigation and data curation; Dmytro Gospodaryov: Statistical analysis; writing - review & editing; Volodymyr Lushchak: Conceptualization, writing - review & editing.
